# The Formation of Human Arteriovenous Malformation Organoids and Their Characteristics

**DOI:** 10.3390/cells13231955

**Published:** 2024-11-25

**Authors:** Eun Jung Oh, Hyun Mi Kim, Suin Kwak, Chanhoe Huh, Ho Yun Chung

**Affiliations:** 1 Cell and Matrix Research Institute, School of Medicine, Kyungpook National University, Daegu 41944, Republic of Korea; fullrest74@knu.ac.kr (E.J.O.); sarang7939@knu.ac.kr (H.M.K.); suin8349@naver.com (S.K.); 2 College of Medicine, Yonsei University, Seoul 03722, Republic of Korea; 99skychan@gmail.com; 3 Department of Plastic and Reconstructive Surgery, School of Medicine, Kyungpook National University, Daegu 41944, Republic of Korea

**Keywords:** arteriovenous malformations, blood vessel organoid, three-dimensional culture, induced pluripotent stem cells

## Abstract

Arteriovenous malformations (AVMs) are characterized by direct connections between arteries and veins without intervening capillaries, with the concomitant formation of abnormal vascular networks associated with angiogenesis. However, the current understanding of the diagnosis and treatment of AVMs is limited, and no in vitro disease models exist at present for studying this condition. In this study, we produced endothelial cells (ECs) in two-dimensional cultures and three-dimensional (3D) blood vessel organoids (BVOs), comparing gene expression profiles between normal and AVM organoids. The normal and AVM organoids were examined via immunofluorescence staining using CD31 and phalloidin. The AVM organoids showed significantly higher expression levels of CD31 and phalloidin than the normal organoids. Genes such as FSTL1, associated with angiogenesis, showed significantly higher expression in the AVM organoids than in the normal organoids. In contrast, the MARCKS gene exhibited no significant difference in expression between the two types of organoids. The capillaries and related CSPG4 genes exhibited the lowest expression in the 3D AVM organoids. Furthermore, hsa-mir-135b-5p, a small RNA related to AVMs, showed elevated expression in AVM tissues and significantly higher levels in 3D AVM organoids. In our study, we were able to successfully establish AVM organoids (hBVOs) containing ECs and mural cells through advancements in stem cell and tissue engineering. These organoids serve as valuable models for investigating disease mechanisms, drug development, and screening potential therapeutic interventions in drug discovery. These findings contribute essential insights for the development of treatment strategies targeting AVMs.

## 1. Introduction

Arteriovenous malformations (AVMs) are the result of a rare condition in which arteries and veins connect directly at high flow rates, bypassing the capillary network and forming new blood vessels and an abnormal network of blood vessels. These malformations around the arteries can arise in various parts of the body due to multiple factors [1]. AVMs are pathologically linked to angiogenesis; however, their cause remains unknown. Without proper treatment, AVMs can lead to adverse effects such as organ dysfunction and cosmetic sequelae [2,3].

The anatomical structure, blood flow, and pathology of AVMs have been confirmed in clinical practice. While various drugs are applied clinically, their definite effects are uncertain. Treatment is challenging due to the lack of disease models, such as an animal model, which has hindered the study of its pathophysiological mechanism [4,5,6,7].

Efficient, manageable, and low-cost methods have been developed to monitor and manipulate AVM cells in two-dimensional (2D) cell cultures [8,9,10]. However, 2D cell culture models have limited application in AVM research because they do not replicate the natural microenvironment or physiology, including cell–cell interactions, morphology reflections, and cell behavior in vivo [11]. Therefore, a three-dimensional (3D) culture system that can accurately mimic the in vivo environment is needed. In recent years, several 3D organoid models mimicking organ structure and function have been developed using human-derived fibroblasts and reprogrammed human pluripotent stem cells (hiPSCs).

These models include hiPSC-derived 3D vascular organoids that facilitate interactions between the ECM and multicellular populations, including vessel walls and endothelial cells. hiPSC-derived 3D vascular organoids also facilitate coordinated functions and hierarchical organization within a biomimetic microenvironment, promoting organoid-specific pathophysiological responses and interactions that resemble natural organs, including intercellular dynamics and activity-dependent adaptation [12,13,14,15,16,17,18,19,20,21]. However, none of these organoids have been developed for AVMs, highlighting the need for drug development using such models to evaluate AVM mechanisms and pathophysiology.

In this study, we aimed to develop blood vessel organoids from AVMs and to compare them with normal blood vessel organoids.

## 2. Materials and Methods

### 2.1. Human Cell Culture

In this study, 24 different human samples were examined. Endothelial cells were obtained from six vessel samples of the tissues of healthy individuals and the tissues of patients with AVM, separately. Additionally, six skin samples were obtained from the tissues of healthy individuals and the tissues of patients with AVM for hiPSC reprogramming, separately.

The samples were selected from a few cases at the vascular anomalies center of a tertiary general hospital. Visceral organ tissues (e.g., brain, gastrointestinal, and pulmonary) were excluded. AVM, as part of a combined vascular anomaly or syndromic AVM (e.g., Parkes Weber syndrome, capillary malformation, arteriovenous malformation, and hereditary hemorrhagic telangiectasia), was also excluded.

For all samples, tissue collected during surgery was immediately placed in sample containers with phosphate-buffered solution (PBS, pH 7.4) and promptly delivered to the laboratory. The study was conducted in accordance with the Declaration of Helsinki and its subsequent amendments. Approval was granted by the institutional review board of Kyungpook National University Hospital (IRB file no. KNUH 2023-04-004-002). Written informed consent was obtained from all patients (Table 1).

#### 2.1.1. Isolation and Culture of Human Fibroblasts

Samples derived from human tissues were exposed to dispase overnight to separate dermal layers and then trimmed to remove fat. Subsequently, they were digested with collagenase type II for 1 h and filtered through a 70 µm cell strainer (Corning^®^, Corning, NY, USA) using DMEM/HIGH (HyClone, Logan, UT, USA).

#### 2.1.2. Isolation and Two-Dimensional (2D) Culture of Human Endothelial Cells (ECs)

The surgical samples of endothelial tissues were rinsed with PBS (LB004-02, Welgene, Gyeongsan, Republic of Korea) and then cut into small pieces. The tissue was cultured in Dispase II (Gibco™, 17105-041, Thermo Fisher, Waltham, MA, USA) at 4 °C for 24 h to separate the epidermis from the dermis. After incubation, the dermal layer was collected and settled in HBSS (14170-112, Gibco, Thermo Fisher Scientific Waltham, MA, USA). The supernatant was removed, and collagenase Type I (4196, Worthington, OH, USA) was added. The sample was incubated at 37 °C, 170 rpm, in a shaking incubator for 1 h. EMB-2 media (cc-3156, Lonza, Basel, Switzerland) was added, and the mixture was passed through a 70 μm nylon filter. After centrifugation (1000 rpm, 5 min), the cells were grown in EMB-2 media and maintained in an incubator at 37 °C with 5% CO_2_.

#### 2.1.3. Reprogramming of Human Fibroblasts and Culture

To generate induced hiPSCs, we reprogrammed human fibroblasts using nonmodified RNA (NM-RNA) in a completely xeno-free culture environment. We used the StemRNA™ 3rd Gen Reprogramming Kit (Cat. No. 00-0076, Stemgent^®^, Reprocell Inc., Tokyo, Japan) and conducted the experiment according to the instructions of the manufacturer. After isolating and culturing human fibroblasts from the tissues of healthy individuals and patients with AVM, we used fibroblasts at 80% confluence for our experiments. The culture medium of fibroblasts was replaced with NutriStem Medium for 30 min before treatment with the NM-RNA reprogramming cocktail. The NM-RNA reprogramming transfer mixture (500 µL) was added to the fibroblasts, which were then incubated overnight in a 37 °C incubator with 5% CO_2_. Subsequently, this process was repeated at the same time daily at 24 h intervals for 4 days. Starting from day 5, the medium was changed daily with fresh NutriStem Medium. The cell length gradually decreased, forming colonies. On day 10–14, when the diameter of the colony was approximately 500 μm or larger, the colony was selected and subcultured on the Vitronectin (VTN-N) (A31804, Gibco, Thermo Fisher Scientific, Waltham, MA, USA)-a-coated dish containing TeSR™-E8™ medium (05990, Stemcell Technologies, Vancouver, BC, Canada). This procedure was performed in a 37 °C incubator with 5% CO_2_. hiPSCs were passaged every 5 days using the Ultra-Pure™ 0.5 M EDTA, pH 8.0 (15575-020, Gibco, Thermo Fisher Scientific Korea, Seoul, Republic of Korea), and rock inhibitor Y-27632 (1254, Tocris, Bristol, UK). The hiPSCs used in this study were kept within 10 passages.

### 2.2. Generation of Blood Vessel Organoids (BVOs)

**Figure 1:**
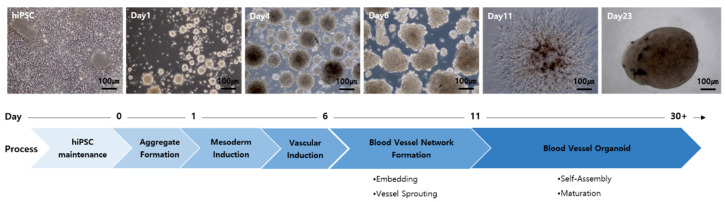
Generation of human blood vessel organoids. Schematic diagram of the protocol.

In this study, we utilized the STEMdiff™ Blood Vessel Organoid Kit (#100-0651, Stemcell Technologies, Vancouver, BC, Canada). hPSCs maintained in E8-medium (05990, Stemcell Technologies, Vancouver, BC, Canada) were detached from the plate using Accutase™ (07920, Stemcell Technologies, Vancouver, BC, Canada) and seeded at 3300–6700 cells/well in a 6-well ultra-low adherent plate (38071, Stemcell Technologies, Vancouver, BC, Canada). The cells were cultured in STEMdiff™ Blood Vessel Organoid Aggregation Medium for 24 h (aggregate formation). Aggregated cells were treated with STEMdiff™ Mesoderm Induction Medium for 3 days (Mesoderm induction). On day 4, vascular induction was initiated by replacing the medium with STEMdiff™ Vascular Induction Medium (vascular induction). On day 6, the aggregates were embedded in a collagen/Matrigel^®^ matrix, including collagen solution (4902, Stemcell Technologies, Vancouver, BC, Canada) and Matrigel^®^ Growth Factor Reduced Basement Membrane Matrix (56231, Corning, Somerville, MA, USA). Aggregates sprouted into vascular networks and matured into stable blood vessels when cultured in STEMdiff™ Blood Vessel Organoid Maturation Medium for 5 days. On day 11, individual blood vessel organoids were isolated from the matrix and maintained in 96-well ultra-low attachment plates (MS-9096UZ, Sumitomo Bakelite, Tokyo, Japan), with a full-medium change performed every 3–4 days (Figure 1).

### 2.3. Immunofluorescence Staining

For staining, the cells were fixed in 4% paraformaldehyde on a rocking platform at a temperature of 25 °C for 1 h. They were then washed twice with PBS and immersed in 0.1% Triton™ X-100 (T9284, Sigma Aldrich, St. Louis, MO, USA) at a temperature of 25 °C for permeabilization. Subsequently, the samples were washed at RT in 0.1% Tween^®^ 20 (P1379, Sigma-Aldrich, St. Louis, MO, USA). Primary antibodies, diluted (1:100) in antibody dilute (003118, Life Technologies, Carlsbad, CA, USA), were then added, namely CD31 (ab76533, Abcam, Cambridge, UK) and OCT4 (ab19857, Abcam, UK), and incubated overnight (>16 h) at 2–8 °C on a rocking platform. Subsequently, the cells were washed three times with wash buffer and incubated with the secondary antibody (goat anti-rabbit IgG H&L Alexa Fluor^®^ 488 A-11008, 1:100, Invitrogen, Waltham, MA, USA) on a rocking platform at room temperature for 3 h in the dark. After additional washing in wash buffer (PBS-T), the samples were counterstained with DAPI and Hoechst nucleic acid stains (H3570, Invitrogen, USA) for 10 min. Following washing in Tween 20, the Vectashield Antifade Mounting Medium (H-1000, Vector Laboratories, Newark, CA, USA) was used, and a coverslip was placed over the samples. Immunofluorescence imaging was conducted using a Leica STELLARIS5 confocal microscope (Leica, DE, Wetzlar, Germany).

### 2.4. Whole-Mount Immunostaining of Organoids

To analyze the overall and internal morphology of organoids, they were fixed in 4% paraformaldehyde on a rocking platform at a temperature of 25 °C for 1 h. After being washed twice with DPBS, the organoids were gently agitated in a gradient series of methanol (50%, 80%, and 100%) at 4 °C for permeabilization. Subsequently, the samples were washed at RT in a series of buffers: 20% dimethyl sulfoxide/methanol, 80% methanol, 50% methanol, PBS, and PBS with 2% TritonX-100. The samples were then blocked in blocking buffer: DPBS with 3% FBS (SH30919.03, HyClone, Logan, UT, USA), 1% bovine serum albumin (A9418, Sigma Aldrich, USA), 0.5% Tween^®^ 20 (P1379, Sigma Aldrich, USA), 0.5% Triton™ X-100 (T9284, Sigma Aldrich, USA), and 0.01% sodium deoxycholate solution (D6750, Sigma Aldrich, USA) on a rocking platform at a temperature of 25 °C for 30 min. After removing the blocking buffer, the primary antibody CD31 (ab76533, Abcam, UK), diluted (1:100) in the blocking buffer, was added. The samples were then incubated at 2–8 °C on a rocking platform overnight (>16 h). Subsequently, the organoids were washed three times with wash buffer, DPBS with 0.05% Tween^®^ 20, and the secondary antibody (goat anti-rabbit IgG H&L Alexa Fluor^®^ 488 A-11008, 1:100, Invitrogen, USA) was added. The reaction took place on a rocking platform at a temperature of 25 °C for 3 h in the dark. After being washed three times with wash buffer, the samples were incubated with Phalloidin (1:20, 12877, Cell Signaling Technology, Danvers, MA, USA) for 3 h in the dark. After further washing with wash buffer, the samples were counterstained with DAPI (Hydrochloride 75004, Stemcell Technologies, Canada) for 10 min and washed with DPBS. The samples were then dehydrated in increasing concentrations of methanol (50%, 80%, and 100%) and incubated with RapiClear^®^ 1.47 (RC147001, SUNJin Lab, Hsinchu City, Taiwan) for clearing at a temperature of 25 °C overnight (>16 h) in the dark. After transferring the organoids to iSpacer^®^ 1.0 mm (IS003, SUNJin Lab, Taiwan), they were observed under a microscope, namely a Leica STELLARIS5 confocal microscope (Leica, DE).

### 2.5. Quantitative Real-Time Polymerase Chain Reaction (qRT-PCR)

The organoids were used to extract RNA using Trizol™ Reagent (15596026, Thermo Fisher Scientific, Watham, MA, USA) and reverse-transcribed into cDNA using RT Premix (EBT-1515, ELPIS, Gwangju, Republic of Korea). qRT-PCR was performed under the following cycling conditions: 95 °C for 10 min, followed by 40 cycles at 95 °C for 15 s and at 60 °C for 60 s in a PCR system (CFX96 touch, Bio-Rad, Hercules, CA, USA) with SYBR Green Supermix reagent (Bio-Rad, Hercules, USA), according to the instructions of the manufacturer. Gene expressions were analyzed using the 2^−ΔΔCT^ method (Table 2).

### 2.6. TaqMan Assay (mir)

The miRNA validations of each group were confirmed using TaqMan advanced microRNA assays (Applied Biosystems, Foster City, CA, USA). All experiments were performed in triplicate. The relative expression levels were normalized using an endogenous control and calculated using the 2^−ΔΔCT^ method (endogenous: has-mir-361-5p, 478056-mir/Has-mir-135b-5p: mir478582) (Table 3).

### 2.7. Statistical Analysis

Data are presented as the mean ± standard deviation of three experimental replicates. One-way ANOVA analysis was used to evaluate differences in miRNA expression levels between groups. Statistical significance was set at a *p*-value < 0.05.

## 3. Results

### 3.1. Identification of Two-Dimensional Cells

**Figure 2:**
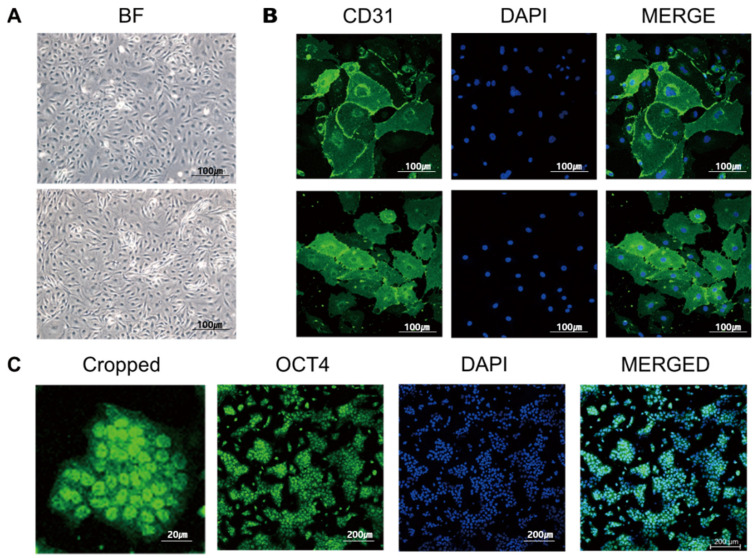
Characterization of two-dimensional (2D) cells. (**A**) Morphology of endothelial cells (top: normal endothelial cells; bottom: AVM endothelial cells; BF: bright field, ×10). (**B**) Immunofluorescence staining of CD31 (green) and Dapi (blue) (top: normal endothelial cells (*n* = 6); bottom: AVM endothelial cells (*n* = 6), ×10). No specific pathological and morphological differences were observed between endothelial cells derived from healthy individuals and patients with AVMs. (**C**) Reprogramming culture morphology progression, resulting from the reprogramming of human fibroblasts (normal skin tissue = 6; AVM skin tissue = 6). Immunofluorescence staining shows that the hiPSCs were OCT4 positive. OCT4 indicates successful reprogramming to hiPSC.

In the 2D culture, no visual differences were observed between normal endothelial cells and AVM endothelial cells under the microscope in BF (Figure 2A). Similarly, immunofluorescence staining of endothelial cells with CD31 did not reveal any expression difference between normal and AVM endothelial cells (Figure 2B). We reprogrammed iPSc using human fibroblasts derived from healthy individuals and patients with AVM to produce blood vessel organoids (hBVOs). Immunofluorescence staining with OCT4—an iPSC-specific biomarker—was performed, confirming their successful production (Figure 2C).

### 3.2. Whole-Mount Immunostaining of Organoids

**Figure 3:**
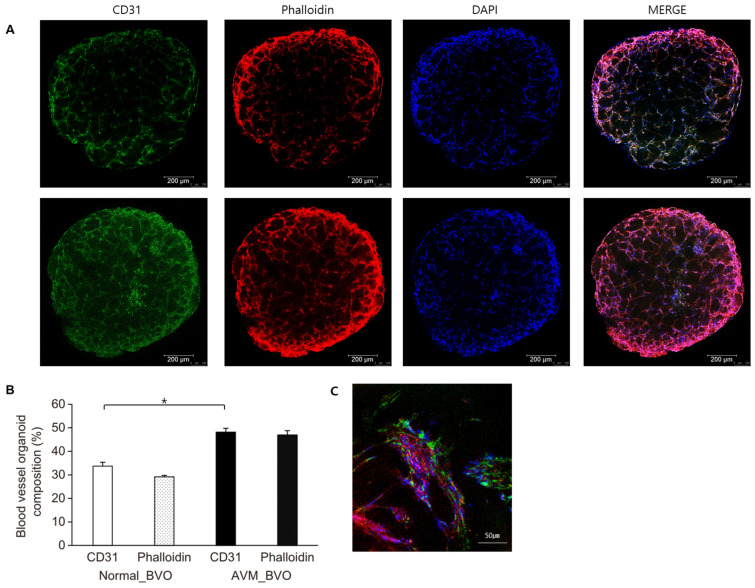
Fluorescence microscopy images of blood vessel organoids differentiated from hiPSCs. (**A**) Normal blood vessel organoids (upper panels, *n* = 6) and AVM blood vessel organoids (lower panels, *n* = 6). Whole-mount staining of BVOs for vascular endothelial networks (CD31, green), vascular smooth muscle networks (phalloidin, red), and DAPI (blue). Scale bars: 200 μm. (**B**) Normal and AVM BVOs contain vascular endothelial networks (CD31) and vascular smooth muscle networks (phalloidin). Quantification of vessels was performed using AngioTool (*n* = 6). Confocal imaging shows the cell types and morphology of blood vessels in vivo, with mesh-type vascular endothelial networks (CD31) surrounded by vascular smooth muscle networks (phalloidin) in hBVOs. AVM BVOs exhibit stronger expression and more complex vascular networks in the lumen and interior than normal BVOs. (**C**) The image shows the tight interaction of vascular endothelial networks (CD31, green) and vascular smooth muscle networks (phalloidin, red) (×40). * *p* < 0.05.

Immunofluorescence staining was performed to determine if the structures represent typical blood vessels. Confocal imaging revealed a mesh-like CD31 expression surrounded by phalloidin. Both normal BVOs and AVM BVOs exhibited vessel-like structures, with visible expression of CD31 and phalloidin. However, CD31, phalloidin, and DAPI exhibited stronger expression in AVM BVOs. Confocal imaging revealed that phalloidin was strongly expressed in the luminal tube, with CD31, a vascular endothelium marker, also showing strong expression within it. Immunofluorescence staining results indicate that AVM BVOs produced from hiPSCs that are derived from AVM fibroblasts exhibit a more complex and pronounced blood vessel organoid composition than normal BVOs produced from hiPSCs derived from normal fibroblasts. This finding is due to the fact that angiogenesis is activated during the development of AVM BVOs, leading to the formation of numerous new blood vessels and the development of abnormal vascular networks. To demonstrate the clarity and precision of the antibody staining of CD31, phalloidin, and DAPI, a cropped image was obtained to expand the region of interest in AVM BVOs. The composition of the organoids was confirmed to be ECs and vascular smooth muscle cells (SMCs) (Figure 3).

### 3.3. Real-Time PCR

The relative expression levels of the three target genes—FSTL1, CSPG4, and MARCKS—selected from differentially expressed genes in 2D normal endothelial cells (2D_Normal), 2D AVM endothelial cells (2D_AVMs), 3D normal blood vessel organoids (3D_Normal), and 3D AVM blood vessel organoids (3D_AVMs) were measured using real-time PCR.

Under normal and AVM conditions, FSTL1 expression was significantly higher in the AVMs than in normal tissues. In the 3D context, FSTL1 expression was significantly higher in the 3D AVMs than in the 2D AVMs (*p*-value = 0.0242)

**Figure 4:**
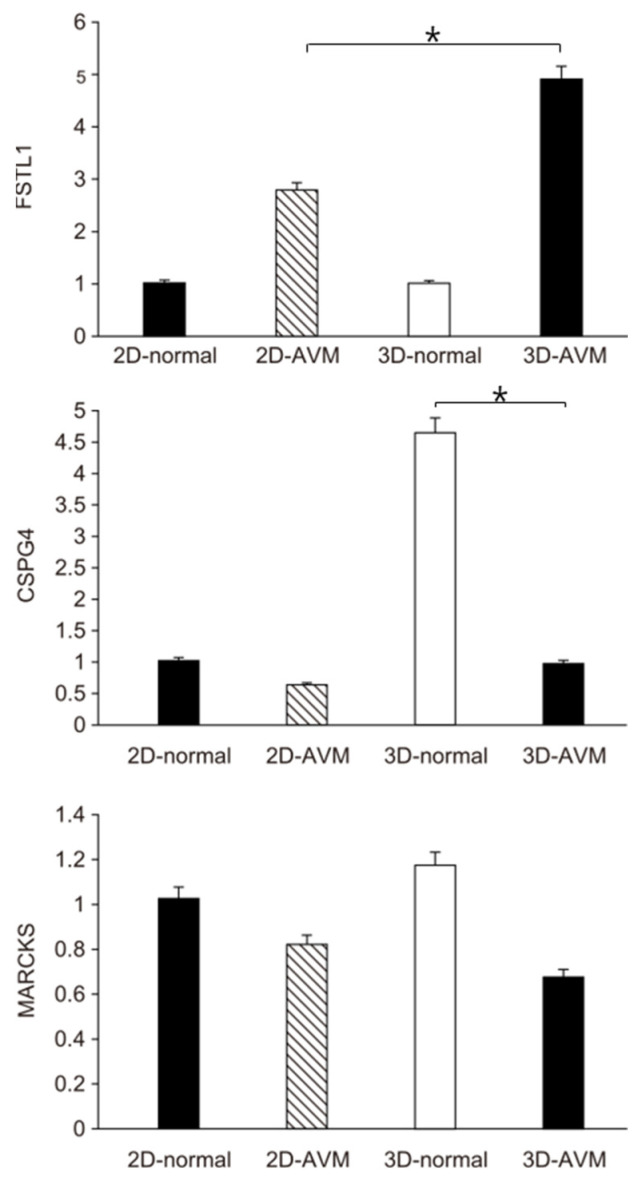
Relative gene expression levels of FSTL1, MARKS, and CSPG4 in 2D normal endothelial cells (2D_Normal, *n* = 6), 2D AVM endothelial cells (2D_AVMs, *n* = 6), 3D normal blood vessel organoids (3D_Normal, *n* = 6), and 3D AVM blood vessel organoids (3D_AVMs, *n* = 6). Real-time PCR was employed to assess the relative expression levels of these target genes selected among differentially expressed genes for 2D normal endothermic cells (2D_normal), 2D AVM endothermic cells (2D_AVMs), 3D normal blood vessel organoids (3D_Normal), and 3D AVM blood vessel organoids (3D_AVMs). In 3D AVM samples, FSTL1 expression was significantly elevated compared with that in the normal samples, indicating significant upregulation in AVMs. CSPG4 expression was lower in AVMs than in the normal controls, especially evident in the 3D normal cells. MARCKS expression was lower in AVMs than in the normal cells and showed similar trends to that of the 2D and 3D models. * *p* < 0.05.

Under normal and AVM conditions, CSPG4 expression was significantly lower in AVMs than in normal tissues. This decrease was particularly pronounced in the 3D state, where CSPG4 expression in AVMs was significantly decreased compared with that in normal tissues (*p*-value = 0.0415). Although no significant difference in MARCKS expression was observed between the 2D and 3D states, MARCKS expression was lower in AVMs than in normal tissues under both states (Figure 4).

### 3.4. TaqMan Real-Time PCR Assays

**Figure 5:**
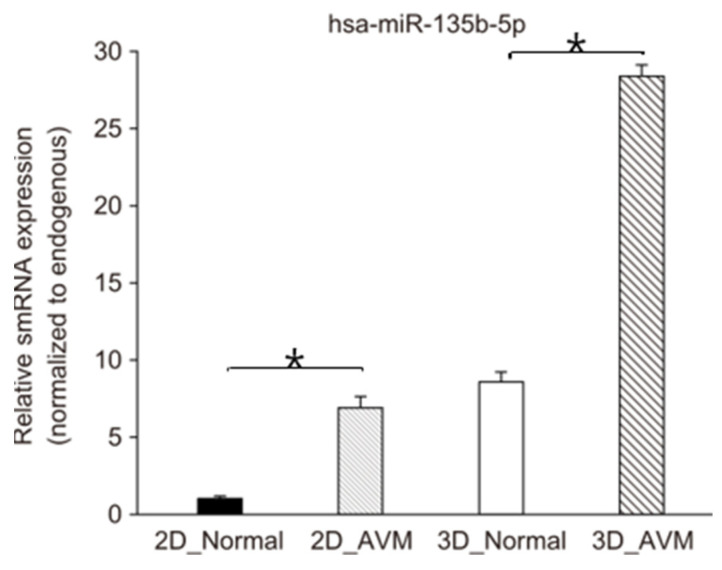
TaqMan real-time PCR assay. The expression level of hsa-miR-135b-5p in 2D normal endothelial cells (2D_Normal), 2D AVM endothelial cells (2D_AVMs), 3D normal blood vessel organoids (3D_Normal), and 3D AVM blood vessel organoids (3D_AVMs). TaqMan real-time PCR revealed that hsa-miR-135b-5p expression levels were significantly higher in 3D than in 2D models. Moreover, AVMs showed higher gene expression levels compared with those in normal tissues, with the highest expression observed in 3D AVMs. * *p* < 0.05.

We conducted a TaqMan real-time PCR analysis of a biomarker involved in the pathophysiology of AVMs, to determine its expression levels in 2D and 3D models [22]. Our results demonstrate that hsa-mir-135b-5p was significantly upregulated in the 3D model compared with that in the 2D model. Both 2D and 3D models showed upregulated expressions in AVMs compared with that in their normal counterparts, with the highest upregulation observed in 3D AVMs. This result confirms that the 3D models more closely replicate the characteristics of AVMs (Figure 5).

## 4. Discussion

AVMs are vascular anomalies characterized by a direct connection to fast-flowing vessels, bypassing the normal capillary network. Their presence results in an abnormal vascular network that can occur in any part of the body, leading to tissue destruction, disturbance of critical structures, and bleeding. Novel treatments for AVMs are urgently needed, emphasizing the significance of early screening and treatment methods. At present, AVMs are treated with surgery and sclerotherapy using various embolic and sclerosing agents. Medications such as thalidomide and sirolimus (rapamycin) are used for symptomatic relief, though their mechanisms of action are not sufficiently understood and are based on clinical experience [23].

One of the limitations in recent studies is the inability to clearly define the initiating and subsequent events of AVM development, which are variable and uncontrollable. Moreover, manipulating AVM tissue at the molecular level and dissecting the underlying signaling mechanisms poses significant challenges. The absence of a reliable disease model to study the mechanisms of and treatment for AVM has hindered the development of clear treatments and therapeutic strategies.

Previous 2D differentiation methods have been highly efficient, manageable, cost-effective, and state-of-the-art for maintaining and differentiating single cells [8]. Nevertheless, 2D cell culture models do not accurately reflect the natural microenvironment or physiology, including cell–cell interactions, and thus differ in morphology and behavior from their in vivo counterparts [11].

Organoids, more physiologically relevant than 2D culture models, are far more suitable for manipulating components, signal pathways, and genome editing, serving as a crucial bridge between 2D culture and animal models. Organoid technology offers a promising avenue for investigating various areas of developmental biology, disease pathology, cell biology, regenerative mechanisms, precision medicine, and drug toxicity and efficacy testing. This technology complements well-established 2D differentiation methods and animal models, offering a more accurate representation of the in vivo environment. Stem cell technology has been a significant field of study for nearly three decades, experiencing remarkable growth and advancement [24,25,26,27].

Since the first successful isolation of mammalian embryonic stem cells in the 1990s, the stem cell technique has evolved significantly. This progress continued with the production of hiPSCs in the early 2000s, leading to the differentiation of pluripotent cells into highly specialized cell types, such as neurons, ECs, cardiomyocytes, fibroblasts, and lung and intestinal cells. Examples of complex tissues and organs successfully modeled in 3D include engineered heart tissues [12], lungs [13], intestinal organoids [14], and so-called “mini-brains” [15]. Stem cell research has now reached new heights, where 3D organoids derived from stem cells that closely mimic the in vivo environment can be produced.

To predict treatment outcomes for patients with various diseases, researchers have employed organoids derived from patient samples before administering treatment to screen drug responses in vitro, serving as diagnostic tools [28,29,30]. The expanding range of experimental applications and organoid culture systems underscores their growing utility and wide applicability, marking a significant advancement in research tools across diverse fields. Therefore, the use of 3D culture systems is warranted to accurately replicate the in vivo environment. Recently, hiPSCs have been used to generate 3D organoids and tissue models that resemble organ structure and function. For instance, a recent publication details the generation of a hiPSC-derived 3D organoid model of the vascular system [16].

In this study, we established AVM organoids from several parts of the body and conducted investigations to develop therapeutic strategies and utilize them for drug screening.

Firstly, we found it challenging to distinguish between normal cells and AVMs in the 2D state using the morphology of ECs and immunofluorescence staining of CD31 (Figure 2A,B).

Human fibroblasts obtained from healthy individuals and patients with AVM were reprogrammed with iPSC. Immunofluorescence staining of OCT4, a specific biomarker of iPSCs, showed that hiPSC was OCT4 positive, indicating that it was successfully reprogrammed with hiPSC (Figure 2C).

hiPSC from normal cells and AVMs are utilized to create hiPSC-derived BVOs, which serve as models for exploring the structural and functional features of blood vasculature. The formation of BVOs involves the differentiation of ECs into blood vessels. Initially, hiPSCs aggregate and undergo induction of mesoderm, followed by vascular differentiation. These vascular-induced aggregates are then embedded in a collagen/Matrigel^®^ sandwich to form a blood vessel structure (Figure 1).

The resulting vascular organoids exhibited lumens, a basilar membrane, and typical tight junctions between ECs, forming a 3D capillary network predominantly consisting of ECs. We identified normal and AVM BVOs as CD31 and phalloidin, and our findings revealed a significantly high blood vessel composition in AVMs. Normal and AVM BVOs are composed of ECs and SMCs, which can be seen to be composed of the luminal structures that contain them (Figure 3).

Genes associated with angiogenesis—FSTL1, CSPG4, and MARCKS—were selected from differentially expressed genes between normal ECs and AVM ECs [9]. FSTL1, known to stimulate angiogenesis in smooth muscle, inhibit smooth muscle cell proliferation via an AMPK-dependent mechanism, and attenuate neovascularization in response to arterial injury, showed significantly increased expression in 3D AVM models [8,23]. CSPG4, present only in arterioles and capillaries, exhibited significantly lower expression in 2D models, with the highest expression in 3D normal models containing arterioles and capillaries. In 3D AVMs lacking capillary networks, the expression of CSPG4 resembled that of 2D models, suggesting that they are closer to true AVMs [11,24,25]. MARCKS, which is implicated in angiotensin II signaling and plays a crucial role in neo-angiogenesis through increased phosphorylation, showed lower expression in AVMs than in normal cells; however, the difference between the 2D and 3D models was not significant, indicating its limited role in the confirmation of organoid formation [26,27] (Figure 4).

A TaqMan real-time PCR analysis was conducted on hsa-mir-135b-5p, a pathological biomarker of AVM identified at the small RNA level. This miRNA promotes Von Hippel–Lindau tumor suppressor (VHL) expression and hypoxia-inducible factor 1-alpha (HIF-1a). Expression levels were significantly higher in 3D models than in 2D models, with the highest expression observed in 3D AVMs. This finding confirms the fidelity of the 3D AVM model in replicating the disease environment [31] (Figure 5).

## 5. Conclusions

In conclusion, we successfully generated six normal blood vessel organoids and six AVM hBVOs containing ECs and mural cells under controlled culture conditions. These organoids represent a significant advancement in stem cell and tissue engineering. The phased formation of BVOs, starting with mesodermal induction and subsequent vascular induction following hiPSC aggregation, provides a novel platform for studying disease mechanisms and developmental processes and evaluating therapeutic efficacy in drug development.

Regarding donor-to-donor variability, some differences in gene expression levels and morphologic features can be observed, but in this study, these differences were not statistically significant when comparing key metrics from donors of different ages and genders etc.

While 3D organoids represent a significant advancement over traditional 2D cultures for modeling complex biological systems, they inherently lack a functional vasculature. This lack of vasculature in 3D organoids is a major limitation, particularly with respect to the efficient delivery of nutrients and oxygen to the innermost cells, in addition to the removal of metabolic waste products. As a result, cells at the core of the organoid often suffer from hypoxia and nutrient deprivation, leading to cell death or altered cell behavior, which can compromise the physiological relevance of the model. In our study, we acknowledge the limitations posed by the lack of intrinsic vasculature in our 3D AVM organoid model. However, by carefully controlling culture conditions and optimizing organoid size, we were able to mitigate some of the issues related to nutrient and waste exchange. Future work will focus on our organoid models, potentially leading to more accurate representations of human arteriovenous malformations (AVMs) and their microenvironment.

## Figures and Tables

**Table 1:** Patient characteristics.

No.	Age	Sex	AVM Status	Sample Type	Location	No.	Age	Sex	AVM Status	Sample Type	Location
1	49	M	None	Skin	Lt. Back	13	6y4m	F	AVM	Skin	Lt. Cheek
2	4y9m	M	None	Skin	Lt. Ear	14	7y3m	F	AVM	Skin	Rt. Ear
3	5y11m	M	None	Skin	Rt. Forearm	15	44	F	AVM	Skin	Rt. Ear
4	59	F	None	Skin	Rt. Inguinal	16	18y4m	F	AVM	Skin	Lt. Chest
5	65	M	None	Skin	Lt. Forearm	17	23	F	AVM	Skin	Lt. Cheek
6	11y6m	F	None	Skin	Lt. Cheek	18	29	M	AVM	Skin	Rt. Heel
7	47	F	None	Blood vessel	Lt. Axilla	19	7y3m	F	AVM	Blood vessel	Rt. Ear
8	75	M	None	Blood vessel	Lt. Axilla	20	27	F	AVM	Blood vessel	Rt. Ear
9	47	M	None	Blood vessel	Lt. Axilla	21	66	M	AVM	Blood vessel	Rt. Trunk
10	29	M	None	Blood vessel	Lt. Thigh	22	52	M	AVM	Blood vessel	Rt. Glabella
11	45	M	None	Blood vessel	Rt. Inguinal	23	29	M	AVM	Blood vessel	Rt. Heel
12	8y3m	F	None	Blood vessel	Rt. Inguinal	24	23	F	AVM	Blood vessel	Lt. Cheek

**Table 2:** Primer sequence for real-time PCR.

Primer Sequence		
FSTL1	Forward sequence	TCGCATCATCCAGTGGCTGGAA
	Reverse sequence	TCACTGGAGTCCAGGCGAGAAT
MARCKS	Forward sequence	CTCCTCGACTTCTTCGCCCAAG
	Reverse sequence	TCTTGAAGGAGAAGCCGCTCAG
CSPG4	Forward sequence	GTCCTGCCTGTCAATGACCAAC
	Reverse sequence	CGATGGTGTAGACCAGATCCTC

**Table 3:** TaqMan primer/probe sequence for RT-qPCR.

Title	Mature miRNA Sequence (5′-3′)
Endogenous	UUAUCAGAAUCUCCAGGGGUAC
hsa-miR-135b-5p	UAUGGCUUUUCAUUCCUAUGUGA

## Data Availability

The data presented in this study are available in the manuscript.
